# Single cell sequencing revealed parathyroid oxyphil cells are involved in osteoporosis under primary hyperparathyroidism

**DOI:** 10.3389/fendo.2025.1603955

**Published:** 2025-05-27

**Authors:** Xinguo Zhang, Ruifeng Bai, Minjuan Li, Zhigang Li, Xian Zhao, Renwei Cao, Shen Tan, Kaiyuan Cheng, Yejun Zha, Xieyuan Jiang, Shuai Lu

**Affiliations:** ^1^ Department of Orthopedic, Shenzhen Hospital (Futian) of Guangzhou University of Chinese Medicine, Shenzhen, China; ^2^ Department of Clinical Laboratory, Beijing Jishuitan Hospital, Capital Medical University, Beijing, China; ^3^ Department of Orthopedic Trauma, Beijing Jishuitan Hospital, Capital Medical University, Beijing, China; ^4^ State Key Laboratory of Environmental Criteria and Risk Assessment, Chinese Research Academy of Environmental Sciences, Beijing, China; ^5^ Department of Orthopedics Trauma, Beijing Jishuitan Hospital, Peking University Fourth School, Beijing, China; ^6^ Department of General Surgery, Beijing Jishuitan Hospital, Capital Medical University, Beijing, China; ^7^ Beijing Research Institute of Traumatology and Orthopaedics, Beijing, China

**Keywords:** primary hyperparathyroidism, single cell sequencing, bone, osteoporosis, parathyroid oxyphil cells

## Abstract

**Objective:**

To analyze the heterogeneity of parathyroid cells between patients with primary hyperparathyroidism (PHPT) osteoporosis and PHPT non-osteoporosis patients.

**Methods:**

Resected parathyroid tissues were collected from PHPT patients of osteoporosis and non-osteoporosis. Single cell sequencing (SCS) to investigate cell types in parathyroid tissue involved in osteoporosis under PHPT. Further cell-cell interaction and communication, pseudotime trajectory analysis, sub-population analysis of parathyroid chief cells and parathyroid oxyphil cells, Gene Ontology (GO), and Kyoto Encyclopedia of Genes and Genomes (KEGG) functional prediction analysis to confirm specific function of parathyroid cells.

**Results:**

Hallmark-IL2/STAT5 and WNT/β-catenin pathways were upregulated in parathyroid cells of osteoporosis patients. Highest interactions and cell-cell communications were enriched in parathyroid cells. Subcluster analysis disclosed overall highest 31.86% CXCL10-PCC parathyroid chief cells, but SPARCL1-OC parathyroid oxyphil cells were higher in osteoporosis patients. Pseudotime trajectory analysis displayed that parathyroid oxyphil cells were in abundance in osteoporosis patients. In total, 281 DEGs involved in kinase activity were identified in osteoporosis patients. Heatmap showed HSPA1A-OC parathyroid oxyphil cells are predominantly involved in numerous and strongest cell interactions. GO and KEGG enrichment revealed PTH, NOTCH, FGF, EGF and CD59 pathways were significantly up-regulated in all parathyroid subpopulations in osteoporosis patients.

**Conclusion:**

Single cell sequencing revealed highest number of parathyroid cells in parathyroid tissue in patients suffering with PHPT osteoporosis. Parathyroid oxyphil cells are predominantly involved in osteoporosis under PHPT.

## Introduction

Primary hyperparathyroidism (PHPT) is an asymptomatic, endocrine malignancy, in 80-90% cases is caused by hypersecretion of parathormone (PTH) due to tumorigenesis in parathyroid glands (1). PHPT causes serious complications in urinary and skeletal system. In PHPT-kidney complication, hypercalciuria, nephrocalcinosis and renal microlithiasis resulted in low glomerular filtration, renal failure and morbidity (2). In PHPT-skeleton complications, excretion of PTH irreversibly damages microarchitecture of trabecular and cortical bones resulted in osteoporotic fractures (3, 4). PHPT-urinary and PHPT-skeletal systems disorders are likely due to mutation in genes involved in regulation of Ca^2+^ in specific cell types (5).

In PHPT-skeletal disorder, reduced bone mineral density (BMD) at cortical and trabecular sites. Insufficiency of vitamin D and excess of plasma fibroblast growth factor 23 (FGF23) are resulted in severe BMD halt (6). If PHPT is not treated immediately, the chances of spine and non-spine fractures are very common (7). In vitamin D deficient PHPT patients, only supplementation of vitamin D is useful in BMD and plasma PTH. Selective estrogen receptor modulator (SERM) and hormone replacement therapy (HRT) in BMD and bone turnover but its non-targeted side effects are very devastating (8, 9). Bisphosphonates enhances BMD and decrease bone turnover but have only applicable in selected BMD patients (10). Calcimimetics causes halt in Ca^2+^ and PTH but not useful in BMD and bone turnover (11). Till date, available treatment of PHPT is parathyroidectomy, if conducted successfully normalizes BMD, bone turnover, and avoids fracture (12).

In recent years, analysis of cell heterogeneity and precise stimulation of specific stem cells has become topic of prime importance. Synovial mesenchymal stem cells (MSC) are progenitors of bone marrow (13). Single cell sequencing (SCS) in combination with lineage tracing and multi-omics has emerged as robust technique to precisely investigate cell heterogeneity and clinical genetic disorders (14). In this study we employed SCS to investigate cell types in parathyroid tissue involved in osteoporosis under PHPT. We further investigated cell-cell interaction and communication, pseudotime trajectory analysis, sub-population analysis of parathyroid chief cells and parathyroid oxyphil cells, Gene Ontology (GO), and Kyoto Encyclopedia of Genes and Genomes (KEGG) functional prediction analysis to confirm specific function of parathyroid cells.

## Material and methods

### Patients and samples collection

A total of 8 PHPT patients (3 non-osteoporosis and 5 Osteoporosis) who underwent parathyroid resection surgery at Beijing Jishuitan Hospital were recruited in our study between January 2021 and February 2022. All patients underwent successful parathyroidectomy and were followed up from the time of diagnosis up to 36.0 months postoperatively. The diagnosis of PHPT was made mainly according to high or inappropriate PTH levels and the presence of hypercalcemia. Patients were included if they met the following criteria: (1) serum PTH level > 65 pg/mL and serum calcium level > 2.75 mmol/L; (2) parathyroid lesion excision performed by experienced physicians in the same department; (3) biochemical and BMD measurement before and after parathyroidectomy; and (4) patients diagnosed with symptomatic PHPT. Patients were excluded if they met the following criteria: (1) incomplete BMD measurements before and after parathyroidectomy or patients who could not be followed up; (2) normal parathyroid gland tissue (i.e. no hyperplasia, adenoma, and parathyroid cancer) diagnosed by histopathological examination after excision of the parathyroid lesions; and (3) serum calcium level remained above the normal range after excision of the parathyroid lesions. Signed informed consent forms were obtained from all subjects before the study. The resected parathyroid tissue samples were collected from all patients during the surgery and stored at -80°C. This study was reviewed and approved by the Institutional Review Board of Beijing Jishuitan Hospital (review batch number 201905–01).

### Single-cell data analysis of parathyroid tissue

In order to perform single cell sequencing data analysis, we followed both automated and manual procedures (15). For data loading and quality evaluation, we employed Seurat v4.0.1 package in R software (16). Following primary standards were adjusted to filter minimum level of cells; (i) total UMI counts < 1200, (ii) gene number < 300, and (iii) mitochondrial gene fraction > 20%. Based on aforementioned standards, in total 51624 cells including 24628 cells of non-osteoporosis and 26996 cells of osteoporosis patients suffering of osteoporosis were selected for further analysis. For data integration, Harmony package v0.1 was employed with default parameters (17). In total, 2500 high differentially expressed genes were identified and top 30 PCs were used for further dimensional reduction analysis. To ensure fidelity of parameters for cell clustering analysis, we determined resolution at 0.2.

### Pseudo-time trajectory analysis

For pseudo-time trajectory analysis in parathyroid cells, Monocle3 v1.2.9 and R package monocle v2.18.0 were individually employed (18), under default parameters. To discover root point, graph learning approach was employed.

### Cell-cell communication analysis

In order to investigate cell communication and interaction, scRNA-seq data was analyzed with the help of CellChat v1.4.0 package in R software (19). In total 1,939 verified molecular interactions were considered in this study from CellChatDB (https://github.com/sqjin/CellChat).

### Sub-population analysis of parathyroid cells

Parathyroid chief cells secrete uncharacterized oxyphil cells in abundance in patients under treatment of hyperparathyroidism (20). We performed in-depth analysis to identify four different types of subpopulations of parathyroid cells in patients suffering with osteoporosis and non-osteoporosis and illustrated in UMAP. Sub-populations are comprised of two types of parathyroid chief cells (S100A13-PCC and CXCL10-PCC) and two types of oxyphilic cells (HSPA1A-OC and SPARCL-OC).

### Functional enrichment analysis

The functional enrichment analysis of Gene Ontology (GO), and Kyoto Encyclopedia of Genes and Genomes (KEGG) was conducted using ClusterProfiler v4.0 (21). We also evaluated the gene signatures scores using UCell v1.3 package (22), singscore v1.2.2 (23), AUCell v1.12.0 (24), GSVA v1.38.2 (25), and irGSEA v1.1.3 (26) in R software.

## Results

### Role of various cell types in hyperparathyroidism

**Figure 1:**
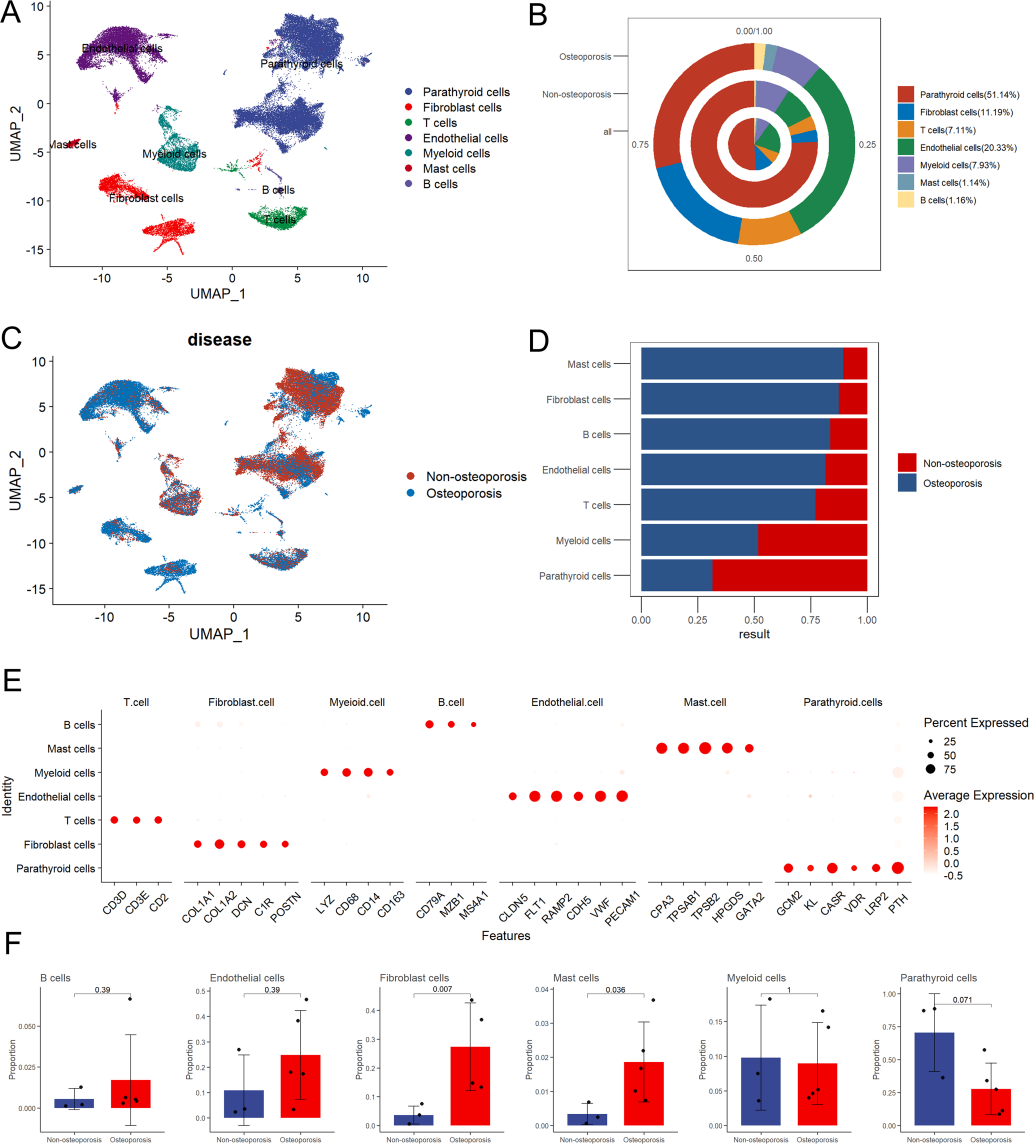
Summarization of cell composition in non-osteoporosis and osteoporosis hyperparathyroidism samples. **(A)** Umap visualization of the cell populations of non-osteoporosis and osteoporosis hyperparathyroidism single cell sequencing dataset. **(B)** Cell proportion pie chart of osteoporosis patients, non-osteoporosis patients and all patients. **(C)** Umap dimensional reduction divided by osteoporosis and non-osteoporosis patients. **(D)** Cell proportion comparison between non-osteoporosis and osteoporosis hyperparathyroidism samples. **(E)** Dot plot of representative cell markers of each annotated cells types. Heatmap of top five markers of each annotated cells types. **(F)** Cell proportion of each cluster. y axis, average percentage of samples in osteoporosis patients and non-osteoporosis patients. Each bar plot represents one cell cluster. Error bars represent ± s.e.m. for 5 osteoporosis patients and 3 non-osteoporosis patients. All differences with P< 0.05 are indicated; two-sided unpaired Mann–Whitney U-test was used for analysis.

In order to reveal relative proportion of various cell types in parathyroid tissue, single cell sequencing data has been manually annotated into seven distinct cell types including parathyroid cells, fibroblast cells, T cells, endothelium cells, myeloid cells, mast cells, and B cells (**Figure 1A**). Among all, parathyroid cells displayed highest share 51.14% which shows their key role in osteoporosis as compared to all other type of cells, followed by 20.33% of endothelial cells, 11.19% of fibroblast cells, 7.93% of myeloid cells, 7.11% of T cells, 1.16% of B cells, and 1.14% of mast cells (**Figure 1B**). Similarly, proportion of parathyroid cells in non-osteoporosis patients was also higher as compared to patients suffering with osteoporosis (**Figures 1C, D**). Furthermore, classical markers expression analysis PTH revealed highest proportion of parathyroid cells among all types of cells, clearly depicted in Umap cluster (**Figure 1E**). Statistical analysis of relative proportion of different cell types in 5 patients suffering with osteoporosis and 3 non-osteoporosis patients were performed (27). We observed highest proportion of parathyroid cells in non-osteoporosis patients, while fibroblast cells and mast cells were significantly higher in patients suffering with osteoporosis (*p* < 0.05) (**Figure 1F**). Expression level of marker genes in each cell types is presented by feature plot and heat map (**Supplementary Figures S1A, B**).

### Cell function and pathway analysis

**Figure 2:**
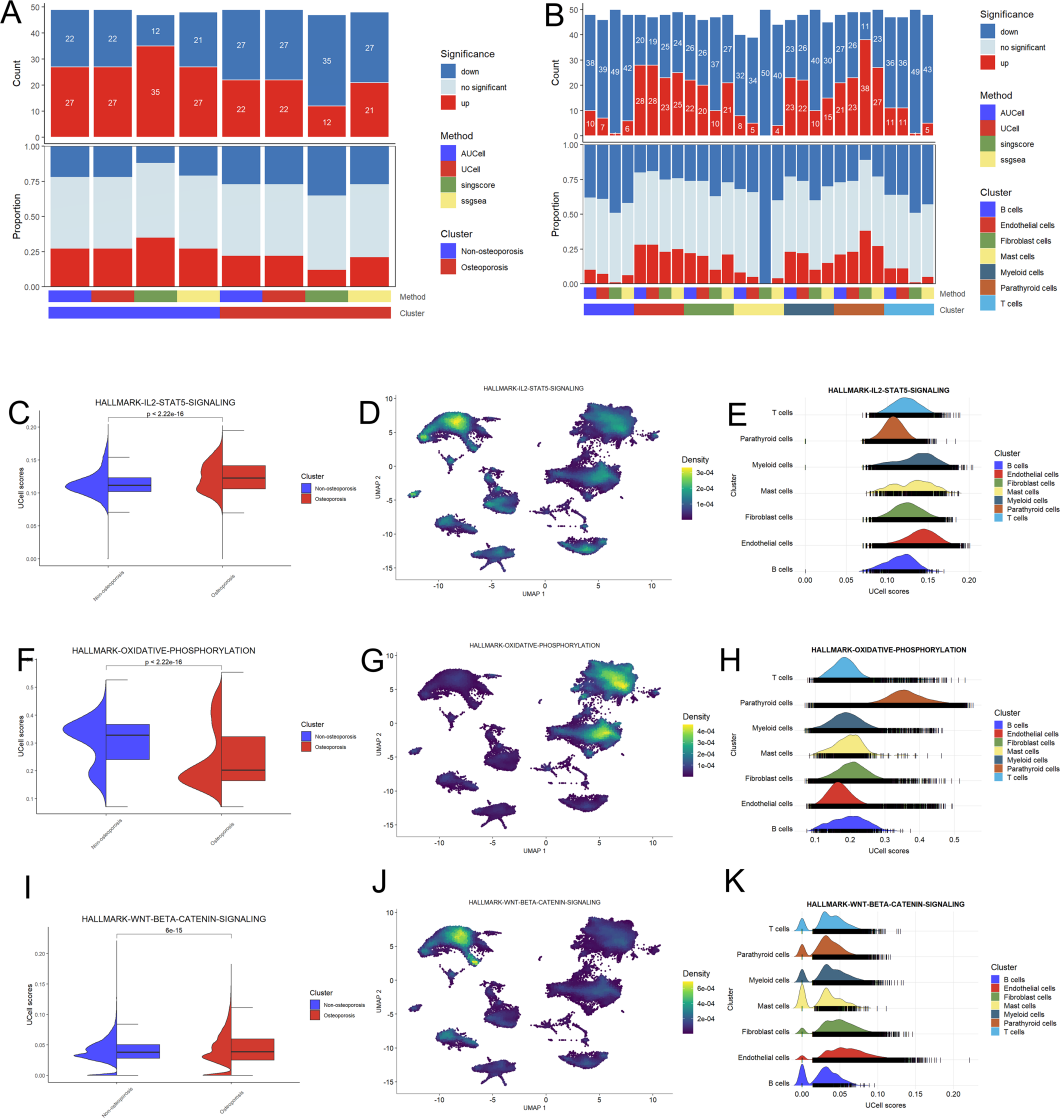
Functional analysis of hyperparathyroidism samples. **(A)** Bar plot of the count and proportion of significant regulation pathway based on four different algorithms for osteoporosis and non-osteoporosis. **(B)** Bar plot of the count and proportion of significant regulation pathway based on four different algorithms for all cell types. **(C-K)** Pathway analysis of three key hyperthyroidism related pathway including IL2-STAT5 **(C–E)**, oxidative phosphorylation **(F–H)** and WNT-beta **(I–K)**. Violin plot showed the difference UCell of these pathway between the osteoporosis and non-osteoporosis patients **(C, F, I)**. Feature plot based on Uamp dimensional reduction show the pathway score distribution across all cell types **(D, G, J)**. Ridge map also show the different pathway score of all cell types in this dataset **(E, H, K)**.

We analyzed pathways and cell functions with the help of following four algorithms; AUCell, UCell, singscore, and ssgsea (**Figure 2A**). We observed no any significant variation in cell function and pathways between patients suffering with osteoporosis and non-osteoporosis. However, these algorisms displayed consistent variable trends on the base of cell types. Comparatively, myeloid, endothelial, and parathyroid cells displayed highest number of significantly up-regulated pathways (**Figure 2B**). These findings indicate that these four cell types are primarily involved in development of hyperthyroidism.

Pathway analysis shows that Hallmark-IL2-STAT5 and WNT-BETA-CATENIN signaling pathways were up-regulated, while oxidative phosphorylation pathway was down-regulated in osteoporosis patients as compared to non-osteoporosis patients (**Figures 2C, F, I**). Further investigation revealed that parathyroid, endothelial, fibroblast, and myeloid cells are involved in these variations (**Figures 2D, E, G, H, J, K**). These evidences further endorsed adaptability of these four types of cells, which probably play key role during pathophysiology of hyperthyroidism.

### Cell-to-cell interaction and communication analysis

**Figure 3:**
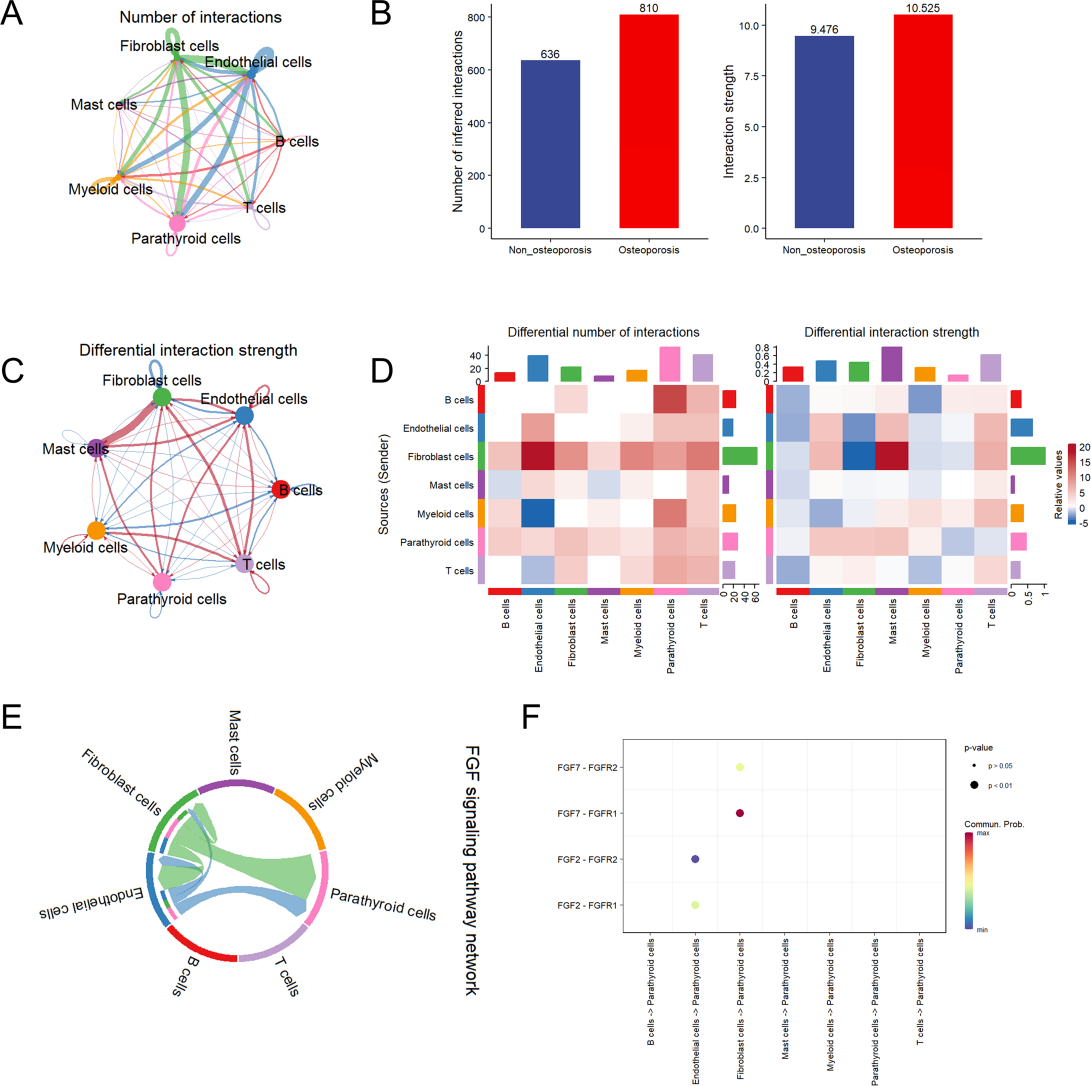
Cell communication analysis of parathyroid cells. **(A)** String plot of aggregated cell-cell communication network in parathyroid sample. **(B)** Number of interaction comparison between non-osteoporosis and osteoporosis patients. **(C)** Interaction strength comparison between non-osteoporosis and osteoporosis patients. **(D)** Differential interaction strength comparison between non-osteoporosis and osteoporosis patients. **(E)** FGF signaling pathway network of all cell types. **(F)** Enriched receptor ligand pairs between parathyroid cells and other cell types in FGF pathway.

The cell function analysis was in agreement with cell communication analysis and *vice versa*. Among all cell types, highest interactions were observed among fibroblast, endothelial, parathyroid and myeloid cells (**Figure 3A**). Specifically, endothelial and fibroblast cells were in communication with parathyroid cells by secreting cytokines. The strength and number of cell interactions were higher in patients suffering with osteoporosis as compared to non-osteoporosis (**Figure 3B**). Moreover, differential interaction strength analysis revealed that fibroblast cells are hub cells playing key role in cell-to-cell interaction between both groups (**Figure 3C**). Further in osteoporosis patients, highest differential number of interactions and their strength was observed in fibroblast cells, which are heterogenic cell cluster and secrete highest signaling molecules to communicate with rest of the cells (**Figure 3D**). Fibroblast cells are predominantly involved in secretion of fibroblast growth factor (FGF) to affect parathyroid cells *via* FGF pathway (**Figures 3E, F**). Due to these reasons, patients suffering with osteoporosis differ in clinical symptoms as compared with non-osteoporosis.

### Subcluster analysis of parathyroid cells

**Figure 4:**
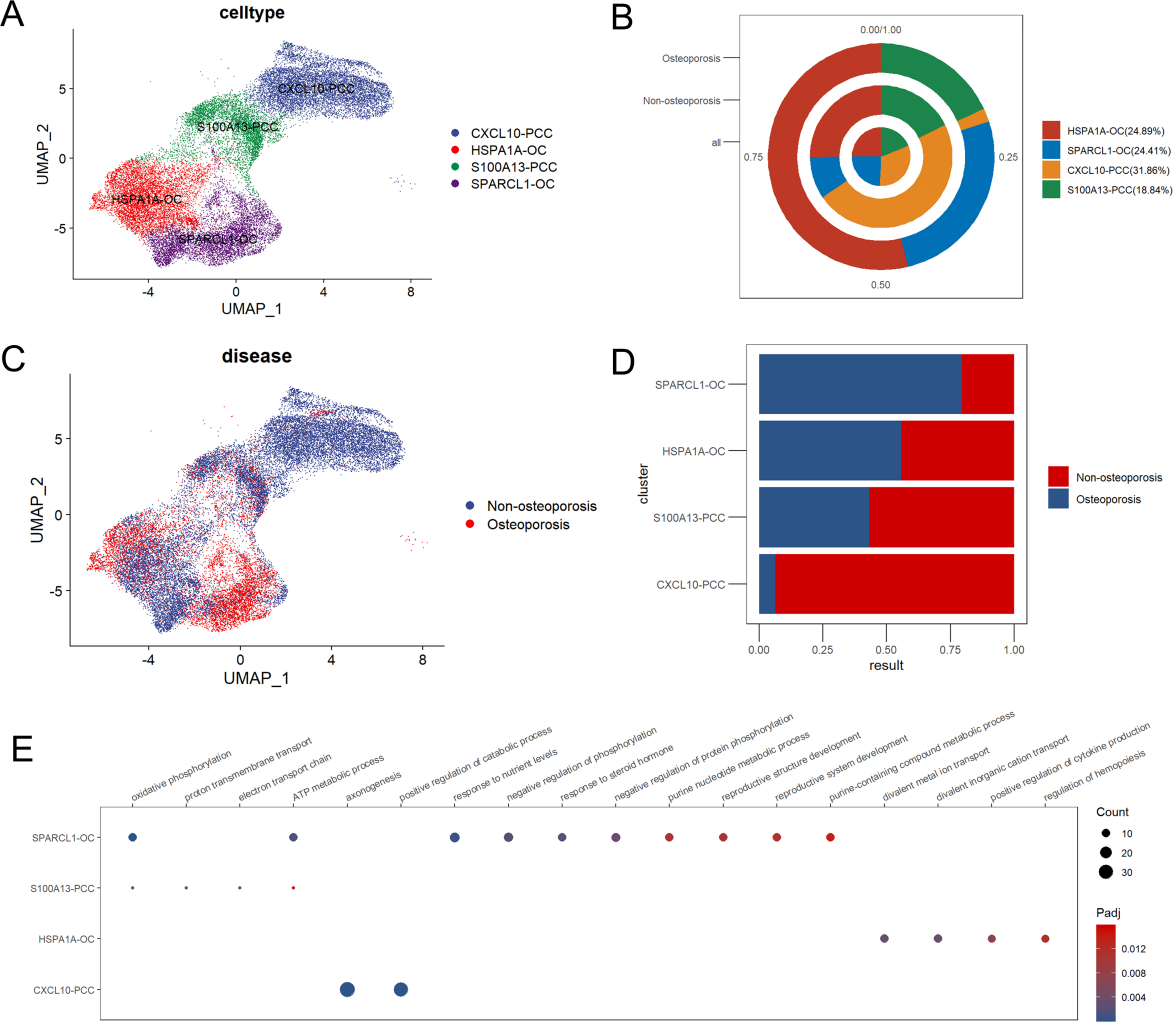
Subpopulation analysis of parathyroid cells. **(A)** Umap dimensional reduction of parathyroid subpopulation **(B)** Cell proportion pie chart of parathyroid subpopulation in osteoporosis patients, non-osteoporosis patients and all patients. **(C)** Umap dimensional reduction divided by osteoporosis and non-osteoporosis patients **(D)** Cell proportion comparison between non-osteoporosis and osteoporosis parathyroid subpopulation. **(E)** Functional annotation of five parathyroid subpopulation.

Subcluster analysis of parathyroid cells revealed highest abundance 31.86% of CXCL10-PCC parathyroid chief cells, followed by 24.89% of HSPA1A-OC parathyroid oxyphil cells, 24.41% of SPARCL1-OC of parathyroid oxyphil cells and 18.84% of S100A13-PCC parathyroid chief cells in parathyroid tissue (**Figures 4A, B**). In patients suffering with osteoporosis, SPARCL1-OC followed by HSP1IA-OC parathyroid oxyphil cells were highly clustered, while in non-osteoporosis patients CXCL10-PCC followed by S100A13-PCC parathyroid chief cells were highly clustered (**Figures 4C, D**). GO enrichment analysis revealed that all four types of cells are functionally independent (**Figure 4E**).

**Figure 5:**
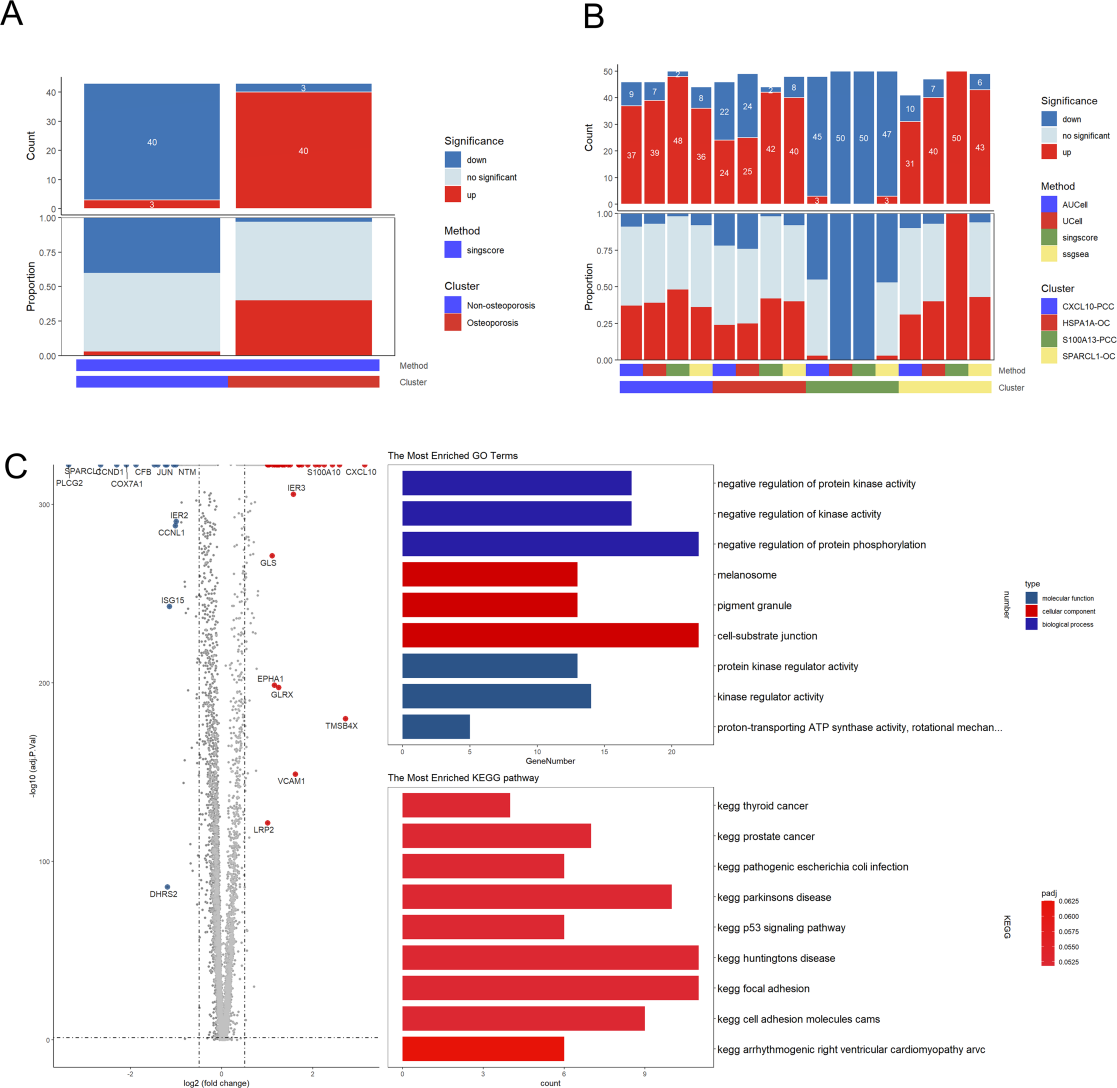
Subpopulation analysis of parathyroid cells. **(A)** Bar plot of the count and proportion of significant regulation pathway of osteoporosis and non-osteoporosis. **(B)** Bar plot of the count and proportion of significant regulation pathway based on four different algorithms for all cell types. **(C)** Volcano plot and functional enrichment analysis of differential expressed genes between osteoporosis and non-osteoporosis. Red points represented the significantly up-regulated genes in non-osteoporosis comparing with the osteoporosis (adjust-p<0.01, logFC>1). While blue points represented the significantly down-regulated genes in non-osteoporosis comparing with the osteoporosis (adjust-p<0.01, logFC<-1).

Functional analysis revealed parathyroid cells in patients suffering with osteoporosis displayed highly up-regulated clusters as compared to non-osteoporosis (**Figure 5A**). We also found that the CXCL10-PCC, HSPA1A-OC, and SPARCL-OC considerably outperformed the S100A13-PCC in terms of up-regulated pathways (**Figure 5B**). In total, 281 differentially expressed genes (DEGs) were identified between non-osteoporosis and osteoporosis parathyroid cells including 177 up-regulated and 104 down-regulated genes (**Figure 5C**). GO analysis revealed that DEGs are highly enriched in regulation of kinase activity. PTH and CD59 were significantly upregulated in all kinds of parathyroid subpopulation in osteoporosis patients (**Figure 5B**). Contrarily, PTHLH was upregulated in non-osteoporosis patients.

### Cell pseudotime and communication analysis

**Figure 6:**
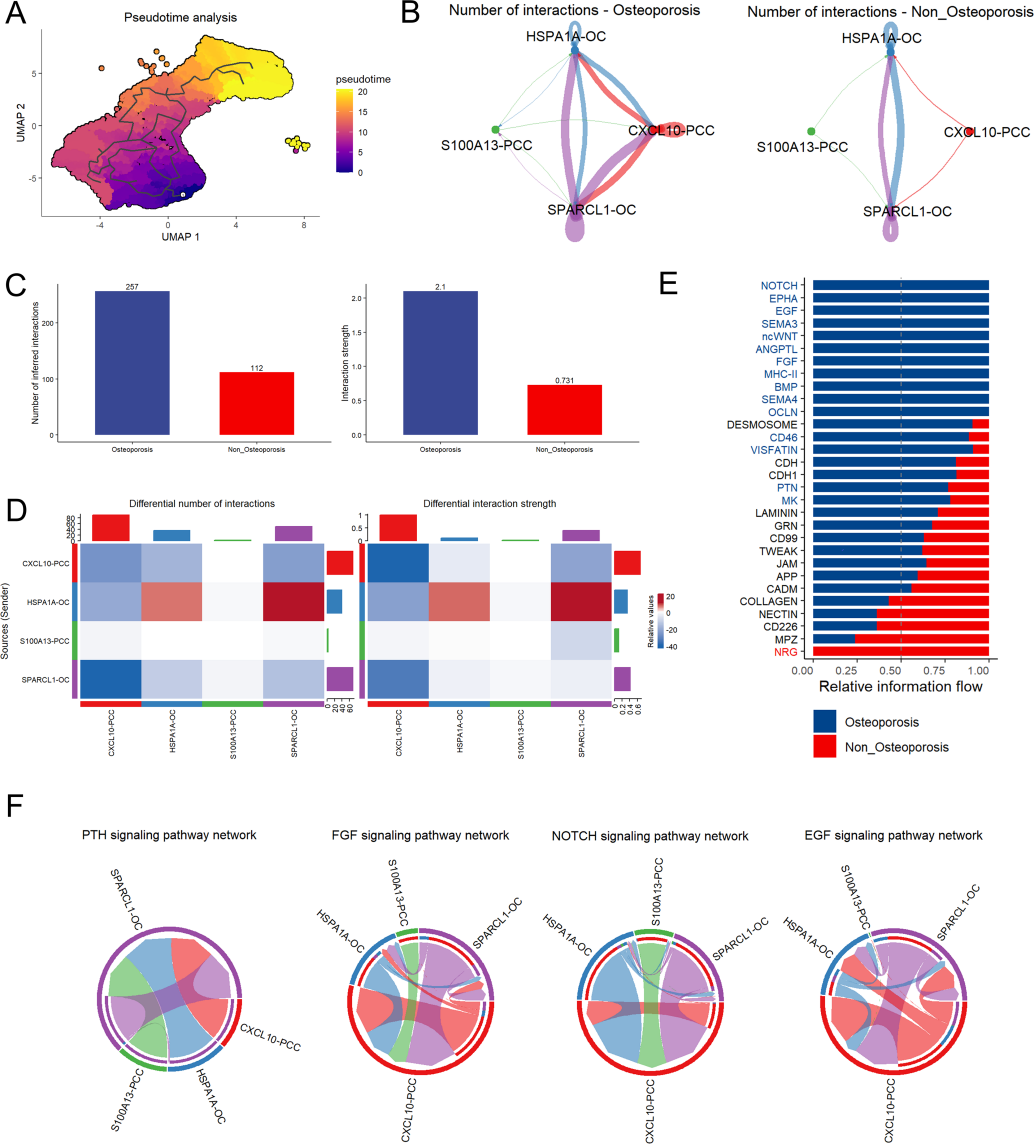
Cell pseudotime and communication analysis of parathyroid cells. **(A)** Pseudotime trajectory analysis of parathyroid cell developmental stages. The tag one was the root point which identified by graph learning. The cell color indicates the pseudotime trajectory (pseudotime) **(B)** String plot of aggregated cell-cell communication network of non-osteoporosis and osteoporosis parathyroid cells. **(C)** Interaction number and strength comparison between non-osteoporosis and osteoporosis patients. **(D)** Heatmap of interaction numbers and strength in different cell subpopulation. **(E)** Differential interaction strength comparison between non-osteoporosis and osteoporosis patients. **(F)** Pathway comparison between non-osteoporosis and osteoporosis patients parathyroid cell subpopulation.

Pseudotime trajectory analysis revealed that oxyphilic cells were more developed as compared to parathyroid chief cells (**Figure 6A**). In both non-osteoporosis and osteoporosis patients, cell communication analysis revealed that CXCL10-PCC, HSPA1A-OC, and SPARCL-OC are predominantly involved in cell-to-cell contact (**Figure 6B**). Compared with the non-osteoporosis, parathyroid cells in osteoporosis patients have significantly large number and higher strength of cell interaction (**Figure 6C**). Specifically, heat map analysis revealed that HSPA1A-OC parathyroid oxyphil cells play key role in large number and higher strength of interactions (**Figure 6D**). Different pathways interaction analysis revealed that osteoporosis patients had higher levels of NOTCH, PTH, FGF, and EGF pathway whereas non-osteoporosis patients had highest level of NRG pathway (**Figure 6E**). According to a PTH pathway interaction analysis, the SPARCL-OC subpopulation is the main source of PTH generation (**Figure 6F**).

## Discussion

Parathyroid hormone (PTH) in is predominantly involved in bone formation (28). Intermittent (hyper or hypo) secretion of serum parathormone (PTH) causes primary hyperparathyroidism (PHPT), which is an asymptomatic endocrinal disorder resulted in osteoporosis of trabecular and cortical bones (1). Single cell sequencing revealed highest number 51.14% of parathyroid cells in parathyroid tissue in patients suffering with osteoporosis, first time reported by us. Parathyroid hormone related classical PTH marker only displayed highest expression in parathyroid cells as shown in Umap cluster, similar findings were reported in *Hypophthalmichthys nobilis* (29). CD59 was also upregulated in in each subpopulation of osteoporosis cells (30). Parathyroid cells are involved in onset and progression of osteoporosis.

Activated signaling pathways such as transforming growth factors (TGF), bone morphogenic proteins (BMPs), fibroblastic growth factors (FGF), wingless type MMTV integration site (wnt) proteins, and transcriptional regulating factors (31) are being employed for identification of true to type cells stem cells. Among all cell types, upregulation of NOTCH, EPHA, ncWNT, ANGPTL, BMP, MHCII, SEMA4, OCLN, FGF, and EGF pathways in parathyroid cells proved their key role in osteoporosis under PHPT, these findings ae in accordance with (32).

Cytokines are secreted by T lymphocytes during exacerbation of inflammatory bone osteoporosis (33). Fibroblast and endothelial cells were in communication with parathyroid cells by secreting cytokines. Notably, these cytokines involved in cell-cell communication can be employed as novel therapeutic strategies against bone loss (34). Parathyroid cells functional analysis in osteoporosis patients displayed highly up-regulated clusters, which shows that osteoporosis parathyroid cells are functionally more activated (35) stated that role of parathyroid oxyphilic adenomas (POA) in onset and progression of PHPT is still controversial. However, in parathyroid tissue samples of patients suffering with osteoporosis, HSPA1A-OC parathyroid oxyphilic cells revealed highest cell-cell communication network, highest number of inferred interactions, and highest differential number of interactions and differential interaction strength. Furthermore, HSPA1A-OC parathyroid oxyphilic cells also revealed highest expression of classical markers associated with PHPT and activated signaling pathway networks Our these findings significantly proved role of parathyroid oxyphilic cells in PHPT, and our these accordance with (36). Parathyroid oxyphilic cells can be used as potential treatment in osteoporosis under PHPT.

## Data Availability

The datasets presented in this study can be found in online repositories. The names of the repository/repositories and accession number(s) can be found in the article/**Supplementary Material**.

## Supplementary material

Supplementary Figure 1 Markers of different cell types in hyperparathyroidism samples. **(A)** Feature plot of representative cell markers of each annotated cells types. **(B)** Heatmap of top five markers of each annotated cells types.

## References

[1] Minisola S Gianotti L Bhadada S Silverberg SJ. Classical complications of primary hyperparathyroidism. Best Pract Res Clin Endocrinol Metab. (2018) 32:791–803. doi: 10.1016/j.beem.2018.09.001 30665547

[2] Verdelli C Corbetta S. Kidney involvement in patients with primary hyperparathyroidism: an update on clinical and molecular aspects. Eur J Endocrinol. (2017) 176:R39–52. doi: 10.1530/EJE-16-0430 27601015

[3] Witteveen J Van Thiel S Romijn J Hamdy N. Hungry bone syndrome: still a challenge in the post-operative management of primary hyperparathyroidism: a systematic review of the literature. Eur J Endocrinol. (2013) 168:R45–53. doi: 10.1530/EJE-12-0528 23152439

[4] Lee G Cotton T Tucci J Aaron RK. Hyperparathyroidism in a fracture population. R I Med J (2013). (2022) 105:34–9. doi: 10.1002/lary.20049 36173907

[5] Romagnoli C Brandi ML. Muscle physiopathology in parathyroid hormone disorders. Front Med. (2021) 8:764346. doi: 10.3389/fmed.2021.764346 PMC8569254 34746197

[6] Lyu Z Li H Li X Wang H Jiao H Wang X. Fibroblast growth factor 23 inhibits osteogenic differentiation and mineralization of chicken bone marrow mesenchymal stem cells. Poultry Sci. (2023) 102:102287. doi: 10.1016/j.psj.2022.102287 36442309 PMC9706642

[7] Eller-Vainicher C Falchetti A Gennari L Cairoli E Bertoldo F Vescini F. DIAGNOSIS OF ENDOCRINE DISEASE: Evaluation of bone fragility in endocrine disorders. Eur J Endocrinol. (2019) 180:R213–32. doi: 10.1530/EJE-18-0991 31042675

[8] Gu P Pu B Chen B Zheng X Zeng Z Luo W. Effects of vitamin D deficiency on blood lipids and bone metabolism: a large cross-sectional study. J Orthopaedic Surg. (2023) 18:1–10. doi: 10.1186/s13018-022-03491-w PMC9826596 36611173

[9] Sas A Tanck E Wafa H van der Linden Y Sermon A van Lenthe GH. Fracture risk assessment and evaluation of femoroplasty in metastatic proximal femurs. An *in vivo* CT-based finite element study. Orthopedic Res. (2023) 41:225–34. doi: 10.1002/jor.25331 35368116

[10] Ginsberg C Ix JH. Diagnosis and management of osteoporosis in advanced kidney disease: a review. Am J Kidney Dis. (2022) 79:427–36. doi: 10.1053/j.ajkd.2021.06.031 34419519

[11] Magbri A El-Magbri M Hernandez PA. Get-up and go: adynamic bone disease in chronic kidney disease patient. Arch Pharm Pract. (2023) 14(1):11–5. doi: 10.51847/suXosREK5t

[12] Nilsson IL. Primary hyperparathyroidism: should surgery be performed on all patients? Current evidence and residual uncertainties. J Internal Med. (2019) 285:149–64. doi: 10.1111/joim.12840 30289185

[13] Liu J Gao J Liang Z Gao C Niu Q Wu F. Mesenchymal stem cells and their microenvironment. Stem Cell Res Ther. (2022) 13:1–10. doi: 10.1186/s13287-022-02985-y 35987711 PMC9391632

[14] Zhang Y Wang J Yu C Xia K Yang B Zhang Y. Advances in single-cell sequencing and its application to musculoskeletal system research. Cell Prolif Basic Clin Sci. (2022) 55:e13161. doi: 10.1111/cpr.13161 PMC8780907 34888976

[15] Clarke ZA Andrews TS Atif J Pouyabahar D Innes BT MacParland SA. Tutorial: guidelines for annotating single-cell transcriptomic maps using automated and manual methods. Nat Protoc. (2021) 16:2749–64. doi: 10.1038/s41596-021-00534-0 34031612

[16] Hao Y Hao S Andersen-Nissen E Mauck WM III Zheng S Butler A. Integrated analysis of multimodal single-cell data. Cell. (2021) 184:3573–87. doi: 10.1016/j.cell.2021.04.048 PMC8238499 34062119

[17] Korsunsky I Millard N Fan J Slowikowski K Zhang F Wei K. Fast, sensitive and accurate integration of single-cell data with Harmony. Nat Methods. (2019) 16:1289–96. doi: 10.1038/s41592-019-0619-0 PMC6884693 31740819

[18] Cao J Spielmann M Qiu X Huang X Ibrahim DM Hill AJ. The single-cell transcriptional landscape of mammalian organogenesis. Nature. (2019) 566:496–502. doi: 10.1038/s41586-019-0969-x 30787437 PMC6434952

[19] Jin S Guerrero-Juarez CF Zhang L Chang I Ramos R Kuan C-H. Inference and analysis of cell-cell communication using CellChat. Nat Commun. (2021) 12:1–20. doi: 10.1038/s41467-021-21246-9 33597522 PMC7889871

[20] Ritter CS Haughey BH Miller B Brown AJ. Differential gene expression by oxyphil and chief cells of human parathyroid glands. Clin Endocrinol Metab. (2012) 97:E1499–1505. doi: 10.1210/jc.2011-3366 PMC3591682 22585091

[21] Wu T Hu E Xu S Chen M Guo P Dai Z. clusterProfiler 4.0: A universal enrichment tool for interpreting omics data. Innovation. (2021) 2:100141. doi: 10.1016/j.xinn.2021.100141 34557778 PMC8454663

[22] Andreatta M Carmona SJ. UCell: Robust and scalable single-cell gene signature scoring. Comput Struct Biotechnol J. (2021) 19:3796–8. doi: 10.1016/j.csbj.2021.06.043 PMC8271111 34285779

[23] Bhuva DD Cursons J Davis MJ. Stable gene expression for normalisation and single-sample scoring. Nucleic Acids Res. (2020) 48:e113–3. doi: 10.1093/nar/gkaa802 PMC7641762 32997146

[24] Aibar S González-Blas CB Moerman T Huynh-Thu VA Imrichova H Hulselmans G. SCENIC: single-cell regulatory network inference and clustering. Nat Methods. (2017) 14:1083–6. doi: 10.1038/nmeth.4463 PMC5937676 28991892

[25] Hänzelmann S Castelo R Guinney J. GSVA: gene set variation analysis for microarray and RNA-seq data. BMC Bioinf. (2013) 14:1–15. doi: 10.1186/1471-2105-14-7 PMC3618321 23323831

[26] Chen B Zhou X Yang L Zhou H Meng M Wu H. Glioma stem cell signature predicts the prognosis and the response to tumor treating fields treatment. CNS Neurosci Ther. (2022) 28:2148–62. doi: 10.1111/cns.13956 PMC9627385 36070228

[27] Patil I. Visualizations with statistical details: The’ggstatsplot’approach. J Open Source Software. (2021) 6:3167. doi: 10.21105/joss.03167

[28] Wan M Yang C Li J Wu X Yuan H Ma H. Parathyroid hormone signaling through low-density lipoprotein-related protein 6. Genes. (2008) 22:2968–79. doi: 10.1101/gad.1702708 PMC2577789 18981475

[29] Luo W Wang J Yu X Zhou Y Tong J. Comparative transcriptome analyses and identification of candidate genes involved in vertebral abnormality of bighead carp *Hypophthalmichthys nobilis* . Comp Biochem Physiol Part D: Genomics Proteomics. (2020) 36:100752. doi: 10.1016/j.cbd.2020.100752 33126027

[30] Losappio V Franzin R Infante B Godeas G Gesualdo L Fersini A. Molecular mechanisms of premature aging in hemodialysis: The complex interplay between innate and adaptive immune dysfunction. Int J Mol Sci. (2020) 21:3422. doi: 10.3390/ijms21103422 32408613 PMC7279398

[31] Bhaskar B Mekala NK Baadhe RR Rao PS. Role of signaling pathways in mesenchymal stem cell differentiation. Curr Stem Cell Res Ther. (2014) 9:508–12. doi: 10.2174/1574888X09666140812112002 25116449

[32] Rahman MS Akhtar N Jamil HM Banik RS Asaduzzaman SM. TGF-β/BMP signaling and other molecular events: regulation of osteoblastogenesis and bone formation. Bone Res. (2015) 3:15005. doi: 10.1038/boneres.2015.5 26273537 PMC4472151

[33] Yang D-H Yang M-Y. The role of macrophage in the pathogenesis of osteoporosis. Int J Mol Sci. (2019) 20:2093. doi: 10.3390/ijms20092093 31035384 PMC6539137

[34] Horwood N. Lymphocyte-derived cytokines in inflammatory arthritis. Autoimmunity. (2008) 41:230–8. doi: 10.1080/08916930701694766 18365837

[35] De la Hoz Rodríguez Á Muñoz De Nova JL Muñoz Hernández P Valdés de Anca Á Serrano Pardo R Tovar Pérez R. Oxyphil cells in primary hyperparathyroidism: a clinicopathological study. Hormones. (2021) 20:715–21. doi: 10.1007/s12022-015-9378-3 34228313

[36] Baregamian N Sekhar KR Krystofiak ES Vinogradova M Thomas G Mannoh E. Engineering functional 3-dimensional patient-derived endocrine organoids for broad multiplatform applications. Surgery. (2023) 173:67–75. doi: 10.1016/j.surg.2022.09.027 36400581 PMC9939934

